# Association between negative life events through mental health and non-suicidal self-injury with young adults: evidence for sex moderate correlation

**DOI:** 10.1186/s12888-024-05880-3

**Published:** 2024-06-24

**Authors:** Yi Zhang, Li Gong, Qing Feng, Keyan Hu, Chao Liu, Tian Jiang, Qiu Zhang

**Affiliations:** 1 Department of Endocrinology, The First Affiliated Hospital of Anhui Medical University, Hefei, Anhui 230022 China; 2 Department of Maternal, Child and Adolescent Health, School of Public Health, Anhui Medical University, No. 81 Meishan Road, Hefei, Anhui 230032 China; 3 Wuxi Huishan District People’s Hospital, Wuxi, Jiangsu 214187 China; 4 The First Affiliated Hospital, College of Clinical Medicine of Henan, University of Science and Technology, Luoyang, Henan 471003 China

**Keywords:** Non-suicidal self-injury, Mental health, Sex, Negative life events, Adolescents

## Abstract

**Background:**

Non-suicidal self-injury (NSSI) has exhibited an increasing trend in recent years and is now globally recognized as a major public health problem among adolescents and young adults. Negative life events (NLEs) are positively associated with NSSI. We sought to explore (1) whether sex plays a role in the risk of NLEs leading to NSSI and (2) the role played by mental health (MH).

**Methods:**

We adopted a multi-stage cluster sampling method to select college students across four grades from May to June 2022. Generalized linear models were used to evaluate the relationships between NLEs, sex, MH and NSSI, presented as incidence-rate ratios (RRs) with 95% confidence intervals (CIs). We examined the complex relationship between these variables using the PROCESS method for moderation analysis.

**Results:**

Following the exclusion of data that did not meet the study requirements, data from 3,578 students (mean age: 20.53 [± 1.65] years) were included. Poisson regression results indicate that high-level NLEs (RR = 0.110, 95%CI: 0.047–0.173) are associated with increased NSSI. Furthermore, interaction effects were observed among sex, NLEs and NSSI. MH and sex moderated the relationship between NLEs and NSSI.

**Conclusion:**

Identifying risk factors for NSSI is also important when exploring the interaction between NLEs and MH given the potential for NSSI to significantly increase the risk of later psychopathological symptoms and substance abuse problems. In addition, the significance of sex differences in risk factors for NSSI should be determined. This study evaluated how the impact of NLEs on NSSI can be reduced among adolescents from multiple perspectives.

## Background

In recent years, economic losses caused by mental illness have been gradually increasing. Compared with symptoms such as depression and anxiety, non-suicidal self-injury (NSSI) has not received sufficient attention. NSSI refers to inflicting physical harm upon oneself without suicidal intention, and is considered a socially unacceptable behavior [1]. NSSI includes various behavioral patterns, such as cutting, burns, beating, scratching, and hair pulling. While distinct from suicidal behavior, it is also a strong predictor of suicide. NSSI continues to cause indelible harm and remains a significant mental health (MH) concern among adolescents worldwide. The Diagnostic and Statistical Manual of Mental Disorders also identifies NSSI as a separate behavioral category and lists it as an “item for further study.” A meta-study revealed that the reporting rate of NSSI (including individual NSSI) among adolescents was 17.2% [2]. Lim et al. estimate the reporting rate to be approximately 19.5% (95% CI = 13.3–27.6% from global literature data) [3]. Another adverse effect of NSSI is that it maintains a “trajectory” into adulthood and increases psycho-behavioral problems during this period [4, 5]. One study estimated the lifetime prevalence rate of NSSI to be 4.86% [6]. In general, the current epidemic of NSSI worldwide is a serious concern [7]. NSSI has evolved into a major public health problem that has endangered the physical and MH of Chinese adolescents. Moreover, NSSI is also linked to suicide, an ongoing social health problem that can lead to further fatal behavior.

If the continued development of NSSI is to be promptly controlled, key questions such as the factors influencing NSSI, its duration, and measures to safeguard those affected by it should be addressed. The factors influencing NSSI in children and adolescents are complex and it is associated with many risk factors. Adolescent’s NSSI is a social phenomenon influenced by various factors that can promote, maintain, or inhibit its occurrence. Therefore, what causes the occurrence of NSSI is worthy of in-depth investigation. As a comprehensive variable encapsulating multiple social factors, negative life events (NLEs) can promote physiological and psychological changes by precipitating shifts in chronic stress states [8]. Adolescence and college years represent critical periods in an individual’s psychological development and life transitions. Students embark on a new, independent phase of life separate from their families and may encounter a series of negative events, which may encompass financial difficulties within families, strained relationships with teachers, experiences of discrimination and rejection from peers, and disruptions in familial dynamics. Furthermore, academic setbacks can affect college students’ daily lives and psychological well-being [9]. Therefore, evaluation of the correlation between NLEs and NSSI and its potential mechanisms from a multidimensional perspective is imperative to promote adolescent MH.

A study exploring rural-to-urban migration in China revealed that child maltreatment and NSSI were associated with family socioeconomic status [10]. Moreover, family environmental factors such as socioeconomic status and family neglect can influence adolescents’ NSSI [11–13]. In addition, using MH, stressful life events (SLEs), and NSSI as the mediating factor, exposure variable, and outcome variable, respectively, research has documented that MH mediates the impact of SLEs on NSSI [14]. Other studies have also established that psychological variables mediate the effect of NLEs on NSSI. All these findings illustrate certain issues. As NSSI can be influenced by multiple factors [15, 16], clarifying the relationship between these factors is particularly important.

Although NLEs have been identified as a risk factor for psychopathology, some studies have not observed a dose–response relationship; this indicates that not everyone who experiences negative events has MH problems and that other factors may be at play. Many causal factors of NSSI remain unexplained by known risk factors, and the overall burden of NSSI underscores the importance of new potential risk factors. To identify potential mechanisms supporting the relationship between NLEs and NSSI, understanding the complex associations or mechanisms beyond simplistic explanations is essential. Research has explained the causes of NSSI using the Four-function Model, which consists of personal, interpersonal, negative, and positive factors [17]. Many factors from multiple levels of socioenvironmental variables may also influence the processes leading to these differences, thus providing a theoretical framework to explain the role of MH in NSSI [16]. Moreover, adopting a life-course epidemiology perspective is helpful to understand the etiology of NLEs concerning physical and MH. The inclusion of early adverse experiences, such as childhood abuse, in public health research topics is particularly significant for health promotion and disease prevention in society.

Overall, the epidemiological literature to date suggests that exposure to NLEs is potentially associated with behavioral and MH problems in adolescents. In addition, variables associated with NLEs such as adverse childhood experiences (ACEs) may increase the risk of NSSI through MH-mediated processes, which are critical for the development of health risk behaviors (HRBs) [18]. These problems can occur regardless of age and geographical culture. According to Meg and Bouchard, age and gender have a strong effect on most psychological and physiological variables [19]. A literature review reveals that the mechanisms underlying the role of sex and MH status in adjustment are stable [17]. While the moderating role of sex with respect to the relationship between NLEs and MH remains relatively understudied, a study indicates that sex does not moderate this relationship [20]. Contrary to the protective advantages associated with sex, negative MH is believed to significantly exacerbate MH problems [6] and stimulate stress reaction mechanisms in the association between NLEs and depression [9]. A series of perspectives, such as those related to diathesis-stress models, shed light on how MH perpetuates the negative effects of NLEs and contributes to the occurrence and recurrence of psychological problems such as depressive symptoms [21]. This suggests that individuals with a history of trauma are more responsive to MH and susceptible to NSSI [6, 22]. Furthermore, the strong correlation between depression, ACEs, and adverse life events among students is well-documented worldwide [23–25]. NLEs can induce psychological distress, even extending to physical discomfort, thereby eliciting negative emotions in teenagers. Individuals exposed to severe NLEs or those unable to cope with setbacks are at risk of developing anxiety and depression, as well as maladaptive behaviors [9]. While the independent effects of sex and NLEs on depression have been reported [26], further correlations require clarification. MH remains an important factor that cannot be disregarded, with research indicating certain biological and behavioral markers meeting the criteria for potential endophenotypes of suicidal behaviors, including early-onset depression [27].

The aforementioned studies affirm the moderating role of NSSI in the relationship between NLEs and MH; however, the potential for sex differences to moderate the relationship between NLEs and NSSI is not yet clarified. The psychological mechanism underlying these associations remains unclear. Moreover, the relationship between NLE exposure and sex differences in the short-term outcomes of NSSI during adolescence has received limited research attention. A more comprehensive understanding of these complex relationships may help in better controlling the occurrence of NSSI. Therefore, this line of research within the specific context of college students needs to be reinforced. The present study investigates the prevalence rate of NSSI among college students and observes the influence of some potential factors on NSSI caused by NLEs. Additionally, it provides empirical evidence for research into the roles of sex, MH, and their impact on NSSI. By integrating the findings of previous research, this study proposes a systematic and comprehensive adolescent MH screening and prevention program, which can serve as a strong basis for establishing relevant guidelines on adolescent MH prevention techniques or expert consensus. Therefore, this study (1) examines the correlations between MH, sex, NLEs and NSSI and (2) investigates the moderating effects of sex and MH in the relationship between NLEs and NSSI among Chinese college students.

## Methods

### Study design

This cross-sectional study was conducted among a population of college students, following the methodology described in previous studies [28, 29]. The surveyed schools adopted cluster sampling, and students of all grades and majors were included. A total of 3,600 college students aged 15–26 years were recruited through an electronic questionnaire survey. Participants were then asked to complete an anonymous questionnaire, which was submitted through mobile phones upon completion. Furthermore, a supervisor was present at the survey site to ensure quality control. The survey was conducted between May and June 2022, and 22 participants were excluded after the survey because they were unwilling to answer the questionnaire or were absent from class. In other cases, missing data (missing values greater than 5%) or obvious errors led to exclusions [30–32]. The design and data collection procedures were approved by the Ethics Committee of Anhui Medical University, and all methods were performed in accordance with the relevant guidelines.

### Exposure

#### Negative life events

In this study, NLEs were assessed using the Adolescent Life Events Scale (ASLEC) developed and revised by Liu et al. The ASLEC consists of 27 items [33], each rated on a scale from 1 to 5 (1 = no impact, 2 = mild, 3 = moderate, 4 = severe, 5 = very severe). Higher total scores mean a greater impact of NLEs risk.

### Mental health

The Depression Anxiety Stress Scales – 21 (DASS-21) was used to evaluate students’ psychological problems. This is a valid and reliable tool that accurately measures depression, anxiety, stress, and other symptoms. The DASS-21 comprises three subscales, each with seven items. Each item has four response options to reflect the severity of psychological problems (0 = No match, 3 = always). Both clinical and non-clinical samples have been used to assess the reliability of the DASS-21 subscale, with favorable results [34].

### Outcome: NSSI

“Have you intentionally hurt yourself in the past 12 months, but not for suicide?” The questionnaire lists several methods of self-injury: hitting with a fist or palm, pulling hair, hitting a hard object with head or fist, pinching or scratching, biting, cutting, and stabbing. Participants who reported engaging in NSSI were further asked about the frequency and the number of NSSI was taken as the total number, with “five kinds” or more defined as NSSI [35].

### Statistics analysis

Mean, standard deviation, and one-way analysis of variance (ANOVA) were used to describe the distribution of continuous variables, and frequency and percentage of Chi-square tests were adopted to characterize categorical variables. We adopted a generalized linear model to assess the relationship between NLEs, sex, MH and NSSI, expressed in terms of incidence ratio (RRs) and 95% confidence interval (CIs). Moderation analysis was conducted by PROCESS method to explore the relationship between NLEs, sex, MH, and NSSI [30, 36]. The bootstrap method was used to re-sample 1,000 samples, and 95% CI was calculated. All data were analyzed using SPSS (Windows Version 23.0). Following preliminary data sorting, missing data were processed via multiple imputation using SPSS 23.0.

## Results

### General demographic characteristics

After data validation and removal of entries that did not meet the study criteria, the final dataset comprised 3,578 students. The general distribution of the variables concerning depression, anxiety, and NSSI is presented in Tables 1, 2 and 3. Among the participants, 1,745 (48.8%) were women; with an average age of 20.53 (± 1.65) years. Regarding residential areas, 1,130 (31.6%) lived in urban areas, 783 (21.9%) in towns, and 1,665 (46.5%) in rural areas. The prevalence rates of NSSI was 8.5%. NSSI was correlated with having fewer than two friends, while depression and anxiety symptoms were correlated with both a worse family economic status and having fewer than two friends. Additional results are detailed in Table 1.

**Table 1:** Distribution of demographic characteristics on NSSI

Variable	Total (*N*, %)	No (*n*,%)	Have (*n*,%)	t/χ^2^ value
Age		20.54 ± 1.56	20.41 ± 2.36	1.38
Sex				1.52
Male	1833	1667(50.9)	166(54.6)	
Female	1745	1607(49.1)	138(45.4)	
Residential area				2.23
Rural	1665	1534(46.9)	131(43.1)	
Town	783	717(21.9)	66(21.7)	
Urban	1130	1023(31.2)	107(35.2)	
Only child				1.88
Yes	11,280	1069(32.7)	111(36.5)	
No	2399	2205(67.3)	193(63.5)	
Father’s education				5.20
Not clear	128	117(91.4)	11(8.6)	
Below junior high	2100	1939(92.3)	161(7.7)	
High school or technical Secondary school	732	664(90.7)	68(9.3)	
Junior college or above	618	554(89.6)	64(10.4)	
Mother’s education				2.55
Not clear	153	141(92.2)	12(7.8)	
Below junior high	2457	2259(91.9)	198(8.1)	
High school or technical secondary school	552	499(90.4)	53(9.6)	
Junior college or above	416	375(90.1)	41(9.9)	
Family economic status				2.99
Bad or worse	1103	996(90.3)	107(9.7)	
Medium	2242	2064(92.1)	178(7.9)	
Better or good	233	214(91.8)	19(8.2)	
Number of friends				19.67**
1–2	1272	1129(88.8)	143(11.2)	
3–5	1730	1605(92.8)	125(7.2)	
6 or more	576	540(93.8)	36(6.3)	

Notes: * *P* < 0.05, **P < 0.01

### Association between independent variables and adolescents’ psychological and behavioral problems

Among adolescent participants at risk for NSSI, 0.152 times as many had depressive symptoms as did not (95%CI: 0.075 ~ 0.229; *P* < 0.001), 0.135 times more with anxiety symptoms than those without (95%CI: 0.064 ~ 0.206; *P* < 0.001), those with stress were 0.220 times higher than those without (95%CI: 0.098 ~ 0.343; *P* < 0.001), and 0.110 times more with anxiety symptoms than those without (95%CI: 0.047 ~ 0.173; *P* < 0.001). These results are reported in Table 2.

**Table 2:** Multivariable linear regression between independent variables and NSSI

	NSSI						
	*χ* ^2^	RR	se	P	DOF	LLCI	ULCI
Negative life events	11.676	0.110	0.0322	< 0.01	1	0.047	0.173
Depression	14.898	0.152	0.0394	< 0.01	1	0.075	0.229
Anxiety	14.002	0.135	0.0361	< 0.01	1	0.064	0.206
Stress	12.442	0.220	0.0625	< 0.01	1	0.098	0.343

Notes: Model 1: Crude model; Model 2: Controlled for parent educational level, sex, economic level, whether only child, number of friends, residential area, and age

**Table 3:** Association between negative life events and sex, depression and NSSI in adolescents

Variables	NSSI					
	coeff	SE	t value	*P* value	LLCI	ULCI
Negative life events	-0.0783	0.0741	-1.0564	> 0.05	-0.2237	0.0670
Depression	-1.2116	0.5217	-2.3224	< 0.05	-2.2345	-0.1887
Sex	-1.9738	2.3751	-0.8331	> 0.05	-6.6305	2.6830
Int_1	0.0288	0.0073	3.9747	< 0.01	0.0146	0.0430
Int_2	0.0455	0.0501	0.9088	> 0.05	-0.0526	0.1436
Int_3	0.6050	0.3549	1.7046	> 0.05	-0.0909	1.3009
Int_4	-0.0131	0.050	-2.5868	< 0.01	-0.0230	-0.0032

Int 1: negative life events × depression; Int 2: negative life events × sex; Int 3: depression × sex; Int 4: negative life events × depression × sex

**Table 4:** Association between negative life events and sex, stress, and NSSI in adolescents

Variables	NSSI					
	coeff	SE	t value	*P* value	LLCI	ULCI
Negative life events	-0.1739	0.0795	-2.1881	< 0.05	-0.3298	-0.0181
Stress	-1.7399	0.5334	-3.2623	< 0.01	-2.7856	-0.6942
Sex	-3.4358	2.4989	-1.3749	> 0.05	-8.3352	1.4636
Int_1	0.0410	0.0080	5.1246	< 0.01	0.0253	0.0567
Int_2	0.0878	0.0541	1.6231	> 0.05	-0.0182	0.1938
Int_3	0.8121	0.3606	2.2521	< 0.05	0.1051	1.5191
Int_4	-0.0182	0.0055	-0.2922	< 0.01	-0.0291	-0.0074

Notes: Int 1: negative life events × stress; Int 2: negative life events × sex; Int 3: stress × sex; Int 4: negative life events × stress × sex

### Moderation analyses of the relationship between NLEs and MH, sex, and adolescent NSSI

Moderation analyses were performed with NLEs, MH, sex, and NSSI and the results are presented in Tables 3, 4 and 5. The findings reveal a positive association between exposure to NLEs and anxiety, alongside a significant association between sex and anxiety. Moreover, the interaction between NLEs and sex significantly predicted the severity of anxiety. However, concerning NSSIs, the interaction between NLEs and sex did not significantly predict the severity of NSSI. Furthermore, the anxiety × sex interaction was correlated with NSSI. After controlling for educational level, consistent patterns were observed across marital status, total annual household income, ethnicity, and age.

**Table 5:** Association between negative life events and sex, anxiety and NSSI in adolescents

Variables	NSSI					
	coeff	SE	t value	*P* value	LLCI	ULCI
Negative life events	-0.0114	0.0774	-0.1478	> 0.05	-0.1632	0.1403
Anxiety	-0.7682	0.5224	-1.4704	> 0.05	-1.7924	0.2561
Sex	-0.6913	2.4570	-0.2814	> 0.05	-5.5085	4.1259
Int_1	0.0199	0.0073	2.7208	< 0.01	0.0056	0.0342
Int_2	0.0148	0.0526	0.2813	> 0.05	-0.0884	0.1180
Int_3	0.3441	0.3556	0.9675	> 0.05	-0.3532	1.0413
Int_4	-0.0084	0.0051	-1.6303	> 0.05	-0.0185	0.0017

Int 1: negative life events × anxiety; Int 2: negative life events × sex; Int 3: anxiety × sex; Int 4: negative life events × anxiety × sex

## Discussion

### Main findings

The prevalence rates of NSSI in our sample of college students was 8.5%. In our generalized linear model, NLEs were positively correlated with the development of NSSI, which suggests that higher exposure to NLEs is related to increased NSSI. Second, we combined NLEs, sex, and MH and established an interaction effect between NLEs, sex, and NSSI. Consistent with our hypothesis, our findings suggest that MH moderates the relationship between NLEs and NSSI; this relationship is also moderated by sex. The relationship between NLEs, MH problems, and NSSI is complex and may involve the moderating role of sex differences. Our findings also provide a new perspective for preventing NSSI among college students [37]. Therefore, we developed and evaluated a moderation model to clarify the role of multilevel factors, including NLEs, MH, and sex, on NSSI [16]. A more comprehensive understanding of NSSI and its associated factors may be helpful in developing programs and interventions to reduce the occurrence of NSSI.

### Comparison with other studies

NSSI is a common MH problem in adolescents [38]. Attention to NSSI is crucial, as it not only has a high incidence but also serves as a major predisposing factor for suicide and even death. The harm of NSSI is that not only can it affect the lives of teenagers, but that it can also further lead to suicidal behavior. A review of 1,094 articles found that NSSI rates have increased globally from 11.5% to as high as 33.8% [39]. Other studies have documented that NLEs have a significant triggering effect on the day and month of a suicide attempt [12]. Importantly, NSSI is associated with, is a specific risk factor for, and can somewhat increase the risk of suicidal behavior. In addition to imposing a personal burden, self-harm, including NSSI and self-harm with suicidal intent, entails significant costs owing to increased morbidity and mortality. This emphasizes the need to investigate the causes and management of NSSI [17]. Despite the high incidence rate, studies on NSSI seem to be insufficient, thereby suggesting the need to pay more attention to the contributing factors. Several factors have been identified, including NLEs, SLEs, negative coping style, problematic Internet use, sleep disturbance, traumatic experiences, problematic parent–child relationships, and MH problems [10, 40–42]. Observing the interactions between variables in more detail requires us to understand the linkages between them.

Variations in NSSI were observed based on sex [10, 35]. A meta-analysis of 18 case-control and seven cohort studies with a total sample size of more than 55,596 individuals reported that NSSI in adolescents was associated with sex differences and mental disorders [35]. Akin to early exposure to environmental endocrine disruptors, NLEs are considered one of the major psychosocial environmental risks that pose a serious and imminent threat to public health and are identified with the increased risk of adult MH difficulties in the future [43]. Exposure to childhood trauma or multiple accumulations of SLEs may lead to an adaptive overload of the biological stress response, thereby dysregulating its development and/or homeostasis [44]. Since the seminal research on cumulative risk, various social and cognitive domains have also presented support for this concept [45]. Using concepts such as the diathesis-stress model and cumulative risk, research has reported that childhood abuse and neglect can significantly increase the risk of psychopathological symptoms in young people [46]. Previous research has recognized these two factors, and the current work also provides answers that complement previous research. Such studies shed light on how NLEs interact with sex factors with regard to NSSI in MH.

We explored the association between MH, sex, NLEs, and NSSI and derived the following observations. First, patients with major depressive disorder and NSSI had experienced ACEs [35, 37, 47]. The present study clarifies the relationship between NLEs and NSSI and provides insights into how therapeutic interventions targeting NLEs can prevent the occurrence of NSSI [37]. Second, previous studies have established that NLEs and MH are associated with higher NSSI [14, 17], and we observed an association between the two in our study. Our analysis is based on the hypothesis of psychopathological development resulting from the interplay between NLEs and behavioral and MH issues [16]. These findings offer a theoretical foundation for a comprehensive investigation of its role in understanding the relationships between NLEs, MH, and NSSI. Third, the interaction between higher-risk NLEs and higher depression was correlated with NSSI [14]. This finding indicates that exposure to low-risk social environments among adolescents with depression can exacerbate MH problems, including common mood and anxiety disorders, self-harm, and suicidal ideation [14]. These results are similar to those of socio-ecological psychology research, which is a process study and can indicate that specific features of the environment lead to a psychological state that can stimulate the target’s behavior through some mechanism (in the current study, psychological problems). These results are also similar to those of previous studies [48, 49]. Finally, we explore the reasons for the association in terms of its underlying mechanisms. Research has highlighted the importance of considering the possible role of chronic inflammatory states by investigating them as mediating mechanisms in the relationship between NLE and NSSI [50]. In terms of biological mechanisms, the hypothalamic–pituitary–adrenal (HPA) axis and circulating inflammatory cytokines may be involved in part of the process [51]. By constructing a gene–environment interaction model, Ben-Efraim et al. explored the effect of interaction between HPA axis regulatory genes (i.e., candidate genes associated with suicide and its endophenotype) and SLEs on suicidal behavior [44]. Furthermore, NSSI self-efficacy may buffer the effect of NLEs and recent NLEs on NSSI [52]; this finding verifies the associations between NLEs, MH, and sex, and NSSI [49]. NLEs are a more complex etiological marker among the factors that cause NSSI, and its effects appear to vary by type, time, and severity, and are also influenced by gender-related factors [53].

Additionally, we explored the moderating effects of MH and sex [49] and identified an association between MH and NSSI [43, 54]. The potential mechanism at play may involve the cumulative and interacting physiological responses to psychosocial stress (including altered neuroendocrine hormone levels, toxic stress, and allostatic load). These processes can lead to nervous system impairments, such as depression and thoughts of suicide/self-harm [55]. In a conceptual model, negative emotions are proposed as an important factor that causes HRBs [56]. We investigated the relationship between sex, MH, and NSSI from a social environment perspective (specifically considering the influence of NLEs) and established that high NLE is associated with high MH and NSSI. Similarly, NSSI and MH issues increased, depending on sex, among adolescents who experienced a poor social environment. The key mechanisms by which stressors such as NLEs lead to behavioral changes such as NSSI include epigenetic changes [57–59]. A series of animal experiments revealed that early adverse life experiences are more important for the change of apparent inheritance pattern; the same is true of population studies. Moreover, ACEs induce changes in MicroRNAs (miRNAs) function through complex interactions of genes associated with the HPA axis and other neuroendocrine signaling systems, further leading to the development of NSSI [60]. Kang et al. explored the association between BDNF methylation, depression, and suicide; they found that a higher BDNF-promoting methylation status was significantly associated with a history of prior suicide attempts, suicidal ideation during treatment, suicidal ideation at final evaluation, higher suicidal ideation scores, and adverse outcomes of suicidal ideation treatment [61].

The aforementioned findings provide empirical evidence for the relationship between NSSI and MH; further, some theoretical models can support our results [17], such as the empirical avoidance model [54], the emotional cascade model [62], and Nock’s integration model [63]. The latter proposes that NSSI combines two motivations (personal, interpersonal, or social) and may in turn be associated with positive or negative reinforcement events [43]. According to Bandura’s social cognitive theory, the potential social factor influence of MH and resulting NSSI may be impacted by resilience [64]; in other words, the relationship between NSSI and sex provides a new perspective into specific NSSI-related cognitions. Our results corroborate and extend those of previous research. Moreover, to explore the mediating role of MH in NSSI and further verify the moderating role of sex, we adopted multiple social environments to portray adolescents’ social interactions, together with their mediating conditions. We also provide perspectives to investigate the association of NSSI with MH and sex, rather than only NLEs (including failure at school, failure to achieve something important, drug use, and bullying), which have been associated with suicidal ideation [65]. The premise of putting forward this complex relationship is that mediating and regulating effects are crucial in psychological research for exploring psychological behavior problems, and the potential mechanisms can be further discovered. This study starts with NLEs, and after observing its association with NSSI, introduces MH, a mediating variable, and sex, a regulating variable. Different positions and roles of these variables in the model will produce different outcomes. A mediating regulating model is a common model that includes both the regulating and the mediating variable. This indicates that the effect of NLEs on NSSI is influenced by sex, and that sex acts (at least in part) through MH.

### Strengths and limitations

The advantages of this study lie in its multilevel design and a large number of adolescent participants. It also explored the associations between complex variables—including moderating effects—that were designed to examine variables influencing the strength and direction of the prediction–outcome relationship and covariates. The use of recommendation in this study could help identify the interdependence between NLEs and NSSI.

However, the following potential limitations should be noted. The cross-sectional design of this study was unable to observe causal inferences of the association between NLEs and NSSI. Future longitudinal studies are needed to assess potential correlations between variables. In addition, the data were obtained using self-recall reports, which can lead to some degree of recall bias. In addition, a key conceptual question in the current literature on NLEs is how researchers conceptualize and measure NLEs; we did not address these aspects (e.g., heterogeneity of NLEs) in our study. In addition, this study only investigated social and psychological factors and did not explore the influence of other biological factors such as inflammation and methylation. Future research should conduct a critical methodological examination of the underlying mechanisms. Finally, as only some variables were included, future research should consider more psychosocial variables [18].

## Conclusion

We found that exposure to NLEs among college students is universal and occurs during a potentially sensitive developmental period. Poisson regression also revealed that NLE, psychological problems, and NSSI are closely related, which has important implications regarding the etiology of NSSI and MH. An interactive positive association was also observed between cumulative NLEs and sex. Finally, moderation analysis should also focus on the MH of adolescents and explore the differences between genders.

## Data Availability

The datasets generated for this study are available on request to the corresponding author.

## Data availability

The data used and analyzed during the study are available from the corresponding author on reasonable request.

## Abbreviations

ASLEC: Adolescent Life Events Scale
ACE: Adverse childhood experiences
CI: Confidence intervals
DASS-21: Depression Anxiety Stress Scales – 21
HRB: Health risk behaviors
HPA: Hypothalamic–pituitary–adrenal
MicroRNAs: miRNAs
MH: Mental health
NLE: Negative life events
NSSI: Non-suicidal self-injury
RR: Rate ratios
SLE: Stressful life events
